# Rapid and Online Microvolume Flow-Through Dialysis Probe for Sample Preparation in Veterinary Drug Residue Analysis

**DOI:** 10.3390/s24123971

**Published:** 2024-06-19

**Authors:** Hanin Diab, Alexandra Calle, Jonathan Thompson

**Affiliations:** School of Veterinary Medicine, Texas Tech University, Amarillo, TX 79106, USA

**Keywords:** online analysis, microvolume sampling, green chemistry, veterinary drug residue, food safety, automated sample preparation, LC-MS

## Abstract

A rapid and online microvolume flow-through dialysis probe designed for sample preparation in the analysis of veterinary drug residues is introduced. This study addresses the need for efficient and green sample preparation methods that reduce chemical waste and reagent use. The dialysis probe integrates with liquid chromatography and mass spectrometry (LC-MS) systems, facilitating automated, high-throughput analysis. The dialysis method utilizes minimal reagent volumes per sample, significantly reducing the generation of solvent waste compared to traditional sample preparation techniques. Several veterinary drugs were spiked into tissue homogenates and analyzed to validate the probe’s efficacy. A diagnostic sensitivity of >97% and specificity of >95% were obtained for this performance evaluation. The results demonstrated the effective removal of cellular debris and particulates, ensuring sample integrity and preventing instrument clogging. The automated dialysis probe yielded recovery rates between 27 and 77% for multiple analytes, confirming its potential to streamline veterinary drug residue analysis, while adhering to green chemistry principles. The approach highlights substantial improvements in both environmental impact and operational efficiency, presenting a viable alternative to conventional sample preparation methods in regulatory and research applications.

## 1. Introduction

The National Academy of Sciences, Engineering, and Medicine defines chemical sensors as devices or instruments that determine the detectable presence, concentration, or quantity of a given analyte [1]. Sensors should provide data of sufficient sensitivity and selectivity to provide adequate information on the analyte that is germane to the needs of the investigation. Sensors which can monitor data nearly continuously (e.g., high temporal resolution) at a specific point in space with low maintenance and minimal cost are also optimal. While no sensor meets all criteria perfectly, most investigators typically envision portable or hand-held devices being rooted in MOS technology, electronic nose systems, or perhaps smartphone sensing or even wearable sensors [2,3,4,5,6,7,8,9,10,11,12,13,14,15,16,17,18,19,20,21,22]. Chemical separations such as chromatography or capillary electrophoresis are rarely considered within technical discussions of sensor technology. The best-known examples of such technology are integrated microfluidic devices [23,24,25,26,27]. However, if separation-based methods can be interfaced with an automated and online sampling strategy for periodically introducing samples, the outcome will be an analytical system for multi-analyte sensing. 

One concern regarding any separation-based method is ensuring that the analytical sample is adequately prepared prior to analysis to prevent clogging the columns or any adverse adsorption of the sample matrix components onto chromatographic surfaces. For complex samples, such as biological fluids or tissue homogenates, sample preparation can be quite a laborious, expensive, and wasteful process. Only 1–10 microliters of fluid sample is typically needed for sample injection and analysis. However, conventional sample preparation steps (pipetting, filtering, centrifuging, etc.) frequently require volumes of solvent and reagents >1000× higher for simplicity and to maintain analytical precision. This necessitates the creation of significant volumes of solvent waste, which must be disposed of while simultaneously enhancing the demand for laboratory-grade solvent stocks. In addition, serial steps for sample handling often produce significant consumable laboratory waste. Neither outcome contributes to green chemistry practices and environmental stewardship.

The European Union and United States place strict controls on the presence of veterinary drug residues in foods for human consumption. When considering analytical methods for the screening of animal tissues for veterinary drugs, several commonly used sample preparation approaches are often employed [28,29]. One approach is solid-phase extraction (SPE), which utilizes a selective adsorption and elution principle. In the traditional format, analytes are enriched, purified, and separated through adsorption onto a solid stationary (or adsorptive) phase placed within a cartridge. A solvent is typically used to desorb the analyte prior to chromatography analysis. An interesting extension of SPE is termed SBSE or stir-bar sorptive extraction [30,31]. In this technique, a magnetic stir bar is coated with a sorbent phase. Traditionally, the extraction of non-polar molecules using polydimethylsiloxane (PDMS) coatings was its primary application. However, more recently, the application of SBSE has widened to more polar substances or even ions through the development of new coating chemistry. Another solid-phase extraction option is called dispersive solid-phase extraction (DSPE). DSPE disperses solid sorbents directly within liquid samples to perform the extraction. After extraction, the sorbent can be isolated by filtration, centrifugation, or magnetic means [32,33]. One such DSPE technology is known as the quick, easy, cheap, effective, rugged, and safe method (QuEChERS), which was created in 2003 by Anastassiades et al. [34]. QuEChERS is essentially a combination of DSPE and salting-out protein extraction. Regardless of which mode of extraction takes place, significant volumes of solvent are used and disposed of during experiments. 

One improvement with regard to green chemistry principles is solid-phase microextraction (SPME) [35,36]. SPME operates by similar principles, except a coated fiber is used to concentrate the analyte. After a prescribed extraction time, the fiber is removed from either the liquid sample or the sample headspace and inserted directly into a chromatographic instrument for analysis. SPME can minimize or even eliminate the amount of extraction solvent that is necessary for more traditional solid-phase extractions. It is commonly known that solid-phase extraction techniques can improve sensitivity and significantly lower matrix interferences [37,38,39]. SPE, however, requires considerable time and effort and may require special apparatus. In addition, recovery or desorption of the analyte may not be consistent, leading to concerns regarding quantitative performance. SPME fibers may be easily damaged, and the breakthrough of the analyte is always a concern when employing sorbents. 

Conventional liquid–liquid extraction (LLE) can also be used for sample preparation prior to veterinary drug analysis. The LLE method is a quick and easy way to analyze veterinary medications using differential partitioning of an analyte between two immiscible solvents. Comparatively high volumes of organic solvents are used in LLE, which is one of the technique’s drawbacks. Supercritical fluid extraction (SFE) is a more environmentally sustainable method of extracting target analytes from matrices compared to solvent extraction. The most popular supercritical fluid is carbon dioxide (CO_2_), which is useful for extracting weakly polar or non-polar compounds but ineffective for polar compounds [40]. Polar compounds can be extracted by altering the supercritical fluid using a polar solvent (like ethanol and methanol) and/or by adjusting the pressure or temperature [41]. Despite being a greener method, SFE is not used as frequently for the pretreatment of veterinary drug residues due to the high cost of the equipment.

Immunoaffinity extraction (IAE) can purify analytes with high selectivity, because it relies on the specificity of the antigen–antibody interaction [28]. Immunoaffinity microextraction in a packed syringe (IA-MEPS) is a particularly facile method which combines IAE with the filling of a reservoir with a solid adsorbent that is connected to a packed syringe [42]. The disadvantages of IAE are that antibodies are required for the target compound, and because the extraction is selective, multianalyte analysis is not possible. Gel permeation extraction (GPE) is based on separation by molecular size. However, there are not many examples of its application in sample purification or cleanup of veterinary medication residues, since these analytes are typically too small to fall within the fractionating range of separation. Clearly, no approach to sample preparation meets the requirements of all analyses, and significant further research is needed to improve the green nature of sample processing steps. 

In this manuscript, we describe an online dialysis extraction method for automated sample preparation prior to liquid chromatography and mass spectrometry. The approach offers three main advantages compared to traditional sample preparation methods. First, the method only consumes microvolumes of reagents, using collected sample more efficiently and, more importantly, minimizing chemical waste and consumption. Second, the removal of cellular debris, particulates, and large molecules occurs automatically through the use of the sensor probe. No costly/waste-generating filtration or time-consuming centrifugation steps are required. Third, the probe is compatible with automated/robot sampling, potentially allowing high-throughput analysis to occur. Such potential for automation does not generally exist when preparing samples through SPE, SPME, or LLE. In the text which follows, we describe the approach, validate the method by conducting a performance evaluation using 45 tissue samples, and demonstrate the method’s utility by screening for the presence of veterinary drug residues in several food products. The probe has substantial promise for reducing chemical waste/use associated with analysis while streamlining and simplifying sample preparation prior to analysis. 

## 2. Materials and Methods

**Figure 1:**
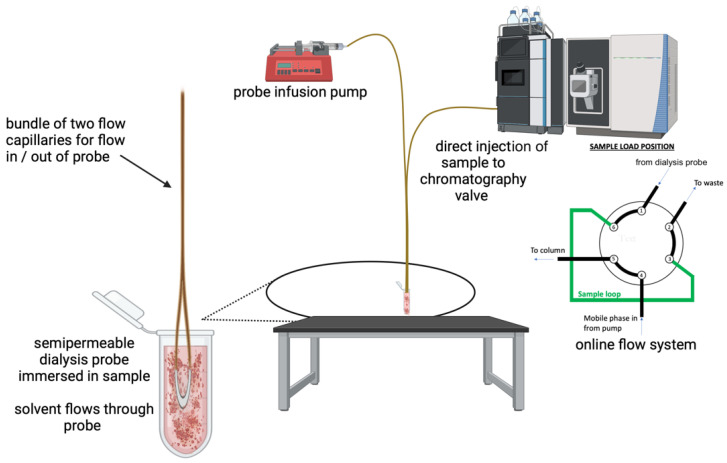
The experimental apparatus for green and automated sample cleanup prior to LC-MS. A fluid pump continuously perfuses a loop-style microdialysis probe at a constant flow rate. The dialysis probe is formed from a semipermeable membrane. Analytes which diffuse through the membrane are swept into the 6-port chromatography injection valve and through the sample loop. Injection and analysis then occur according to standard protocols.

The apparatus used for analysis is depicted in Figure 1. We refer the reader to this figure for a clear explanation of the analysis system.

### 2.1. Microdialysis Probe Construction

Loop-style microdialysis probes were fabricated from regenerated hollow cellulose fiber dialysis tubing (200 µm i.d.; 216 µm o.d.) of 18,000 MWCO. A short section (4–7 cm) of the fiber was clipped using scissors. Then, the fiber was hydrated by soaking in R.O. water, and a 20–30 cm length of 190 µm o.d. × 75 µm i.d. of fused silica capillary was inserted approx. 1–2 cm into the fiber with the aid of a microscope. Then, the membrane fiber was affixed to the capillary tube and sealed with cyanoacrylate glue. This process was then repeated for the additional end of the fiber. Next, the fiber membrane was again hydrated and carefully bent to form a loop geometry. Then, two 2–3 cm lengths of 1/16″ o.d. × 360 µm i.d. Teflon tubing were slid over the inlet and outlet ends of the smaller bore capillary tube at a position of approx. 1 cm and sealed with cyanoacrylate. These sleeves were added to increase the tubing diameter to facilitate fluid connections. Then, two fluid connection tubes (with one end being a NanoViper (ThermoFisher, Bremen, Germany) fitting and the other a 360 μm o.d. fused silica capillary) were obtained. The capillary ends were slid into the Teflon connectors while the NanoViper fittings connected to either the perfusion pump or the chromatography valve. The microdialysis probe was connected directly to the valve to achieve the direct injection into the sample loop, while the probe was submerged in the sample.

### 2.2. Sample Preparation

The analytes for this study were streptomycin sulfate (MP Biomedicals, Santa Ana, CA, USA), oxytetracycline HCl (>95% TCI), ractopamine HCl (Sigma Aldrich, St. Louis, MO, USA), doxycycline HCl (Fisher Scientific, Hampton, NH, USA), phenylbutazone (99+% Thermo Scientific), sulfadimethoxine (>98% TCI), rifampicin (>98% TCI), and Penicillin G (MP Biomedicals) and were used as obtained from the manufacturer. Briefly, standard solutions of each veterinary drug were prepared separately (typically low mg/mL conc.) and subsequently diluted further to prepare analytical standards for analysis. Solvents for chromatography were all LC-MS grade. 

Beef liver (frozen), whole milk, and pet food paste were purchased from a local market. The beef liver was defrosted and blended, adding water, to form a tissue homogenate. The whole milk was analyzed as received. The pet food samples were known to be a homogenate/paste of various food animal organs. The pet food paste was mixed with water to form a homogenate and subsequently analyzed. Based on running these samples alone, we found no detectable analytes to be present in the products. Thus, to prepare samples for analytical testing, it was necessary to randomly spike samples with various veterinary drugs in known quantities to achieve analyte concentrations <1000 ng/mL. The exact concentrations used for each analyte are reported in the data and figures below.

For analysis of foods, a selection of cheeses, butter, milk, cream, and ground meats were obtained from France, Germany, Switzerland, Guatemala, and the United States. In the lab, 1 cc of each sample was placed into a conical vial, and 15 mL of a 50:50 *v*/*v* solution of water and methanol was added. Then, the raw food sample was ground in the solvent to promote extraction and dissolution of any drug residues present. 

### 2.3. LC-MS Conditions

A Thermo Fisher Ultimate 3000 UPLC chromatograph, coupled with the Q-Exactive HF mass spectrometer (Bremen, Germany), was utilized for LC-MS analysis. For separation, a Magic 3 C—18 reversed-phase column (Premier LCMS, USA), 15 cm in length, 75 μm i.d., and with 1.8 µm diameter particles, was employed. The mobile phases consisted of A (water and formic acid (0.1%)) and B (acetonitrile with formic acid (0.1%)). Gradient elution chromatography was carried out at a constant flow rate of 400 nL/min. Initially, the eluent comprised 5% B, and this composition was maintained until t = 2 min. From 2 to 8 min, the percentage of B increased linearly to 70%. Then, between 8 and 14 min., B increased to 95%, and this was maintained until the 18 min mark. At 18 min., the composition returned to 5% B and was maintained until 23 min. to prepare for the next chromatographic run. 

The dialysis probe was perfused using the chromatograph’s loading pump (the probe infusion pump in Figure 1). The solution that was perfused through the probe was a ternary mixture of water (w/0.1% formic acid), a mixture of methanol (75%) and water (25%), and acetonitrile (w/0.1% formic acid). The perfusate composition was held constant as 80% water, 10% of the mixture, and 10% acetonitrile. For the analysis of tissue samples, the flow rate through the microdialysis probe was 2 µL/min. 

The Q Exactive HF Orbitrap mass spectrometer (ThermoScientific, Bremen, Germany) was working under ESI positive mode. For the analysis, the method consisted of a full MS scan at a resolution of 120,000 from *m*/*z* 100 to *m*/*z* 1500 (automatic gain control target of 5 × 10^6^ or 100 ms maximum injection time). For MS2, a resolution of 30,000 was set, with automatic gain control of 2 × 10^5^ and 50 ms maximum injection time and an isolation window of 4 *m*/*z*. The normalized collision energy was set to 30 (arb units). The ESI spray voltage was 2.4 kV for positive ion mode, with an inlet capillary temperature of 270 °C. 

Selected ion chromatograms were generated by filtering MS data for known mass peaks ± 5 m.m.u tolerance. The following MS peaks were selected for each drug: sulfadimethoxine 311.078 Da, oxtetracycline 461.152 Da, rifampicin 823.409 Da, phenylbutazone 309.158 Da, doxycycline 445.158 Da, penicillin G 335.104 Da, ractopamine 302.173 Da, and flunixin 297.0831 Da. The analysis of the acquired data and the extraction of the retention times of the MS spectra and peak areas were achieved using the Thermo Scientific Freestyle software ver.1.7 (Bremen, Germany).

## 3. Results

### 3.1. Chemical Analysis of Veterinary Drug Residues

#### 3.1.1. Chromatography

**Figure 2:**
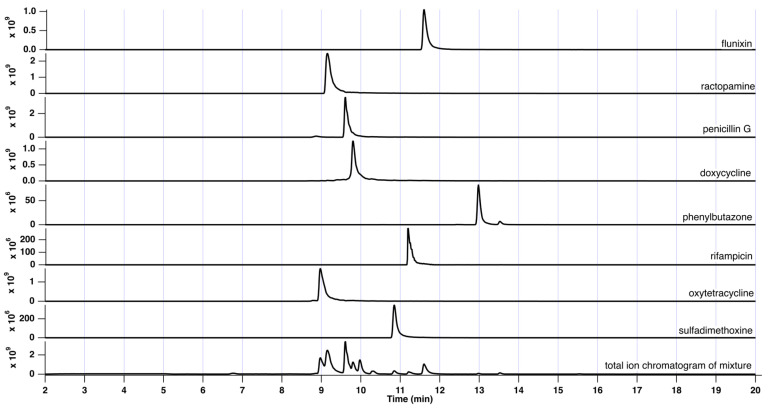
Total ion chromatogram of veterinary drug mixture (bottom trace). Additional traces depict selected ion chromatograms for each drug within a tolerance of ±5 m.m.u.

Figure 2 illustrates selected ion chromatograms for a series of veterinary drug standards and a total ion chromatogram for the mixture (bottom). In addition, Table 1 lists the typical chromatographic performance values observed. 

As reported, veterinary drugs eluted between approx. 9 and 13 min on the reversed-phase column. Peak widths were analyte-specific but generally between 0.25 and 0.5 min at baseline. Thus, the column generated between 6980 and 33,000 theoretical plates for the analytes tested. 

#### 3.1.2. Mass Spectrometry

Table 2 lists the mass spectral peaks observed for the veterinary drugs in the first and second dimensions. Limits of detection (for MS1), as measured through the probe, were in the low ng/mL range for analytes tested. However, no attempts were made to optimize detection limits during this study. Further enhancement in detection may be achieved by pursuing selected (precursor) ion monitoring or the preconcentration of analytes prior to injection on a chromatographic trap cartridge. During MS, the expected molecular ion peaks were observed for all analytes. Following collisionally induced dissociation (CID), characteristic fragment peaks appeared for most veterinary drugs. However, the measurements with rifampicin did not yield extensive fragmentation of the parent ion under the conditions tested. 

The full MS2 spectra of the veterinary drugs tested are provided to the reader as Supplement S1. To identify samples that were positive for drug residue, we only used the chromatographic retention time for confirmation, checking that the peak observed also matched the corresponding molecular ion.

### 3.2. Microdialysis Probe Performance and Recovery

Since microdialysis probes are continually perfused during operation, this experiment represents a non-equilibrium state of mass transport across the membrane, and therefore, the concentration of analyte inside the membrane is generally lower than the analyte concentration outside the membrane. Probe recovery (%R) is the ratio of analyte concentration inside compared to outside the probe, expressed as a percentage:

(1) $$\%R=\frac{analyte\;conc.inside\;probe}{analyte\;conc.outside\;probe}\times 100$$

**Figure 3:**
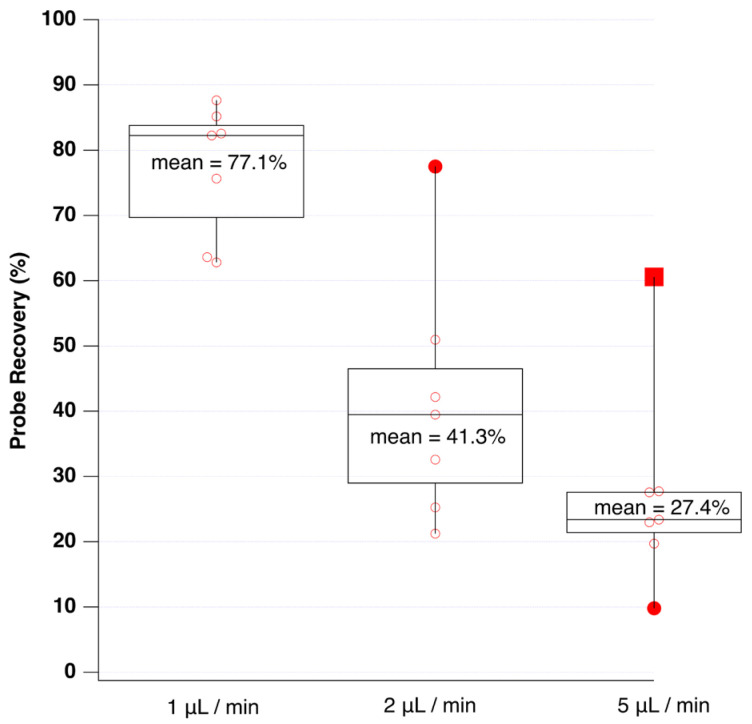
Box-plots of microdialysis probe recovery at 1, 2, and 5 microliter per minute flow rates. Probe recovery is defined as the percentage of the apparent concentration of a drug inside the dialysis probe compared to the concentration outside the probe. The concentration was inferred from the peak area. Data represent the average for all analytes.

Generally, probe recovery increases at lower flow rates, since additional time is allowed for analyte diffusion to occur. Figure 3 reports the observed analyte recovery at probe perfusion flow rates of 1, 2, and 5 μL per minute.

As observed, the average probe recovery was lowest (27.4%) at a perfusion flow rate of 5 microliters per minute. However, the average recovery was observed to increase to 77% at 1 microliter per minute. The only technical disadvantage of low perfusion flows was the additional time required to flush the connecting tubing and fill the LC injection loop completely. 

### 3.3. Veterinary Drug Residue Detection in Mock Samples

**Figure 4:**
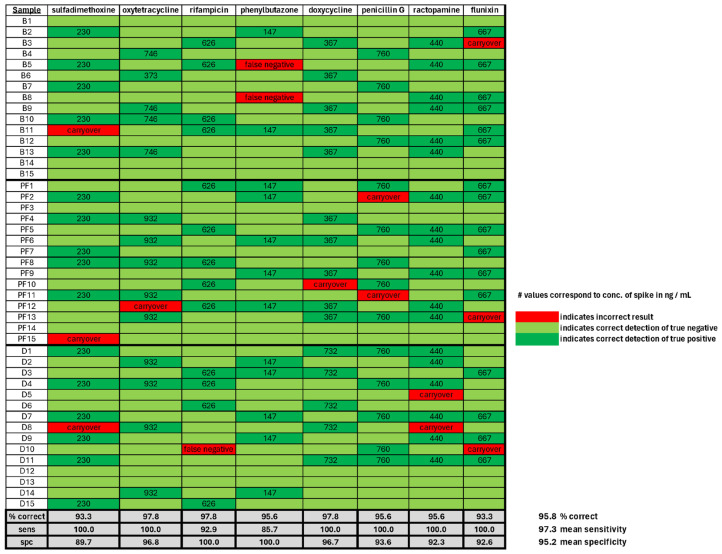
Summary of results for detection of veterinary drugs in spiked animal tissue homogenates. Red—incorrect results, Dark green—correct detection of true positive, Light green—correct detection of true negative. Carryover refers to positive diagnosis perceived to be caused by contamination by previously analyzed sample. Numerical values correspond to concentration of residue spike in ng/mL. Specificity and sensitivity computed via standard approach [45].

To examine whether the low-solvent-consumption probe is effective at detecting veterinary drugs in foods, we obtained food products from a local market that is known to be free of analytes and then randomly spiked drugs into 15 mL homogenates or pureed food samples. Three sample classes were used: beef liver puree (B), pet food puree (PF), and pasteurized milk (D). The pet food sample consisted of ground organs of beef, swine, and poultry. Each sample was then screened for the presence of veterinary drug residues, and the test results were compared with known samples for the computation of sensitivity and specificity. The concentration of analytes used was in the range of 100–1000 ng/mL, which is similar to values previously observed in animal tissues and the maximum regulatory limits for residues present in foods [29,43,44]. Figure 4 reports the results of this analysis. 

The green boxes in Figure 4 represent the correct results, with dark green representing a correct positive diagnosis and lighter green representing a correct diagnosis of a negative result. Nearly 96% of the results yielded the correct diagnosis, with a sensitivity of 97.3% and specificity of 95.2%. The red boxes in Figure 4 correspond to experimental results which were inaccurate. The most frequent incorrect results manifested as false positives, apparently caused by sample carryover from the previous analysis. On at least three occasions, false negative results were obtained. The cause of these results is less clear but could be due to user error in pipetting and/or sample preparation. This analysis clearly demonstrates the ability of the microvolume sampling probe to filter and clean raw samples from animal tissues for direct injection onto a chromatograph and yield accurate diagnoses of the presence or absence of veterinary drugs.

### 3.4. Veterinary Drug Residue Detection in Food Samples

Given the successful demonstration of the mock analysis for drug residue analysis, we next employed the microvolume sampling system for analysis of authentic food samples. A selection of cheeses, butter, milk, cream, and ground meats were obtained from France, Germany, Switzerland, Guatemala, and the United States. In the lab, 1 cc of each sample was placed into a conical vial, and 15 mL of a 50:50 *v*/*v* solution of water and methanol was added. Then, the raw food sample was ground in the solvent to promote the extraction and dissolution of any drug residues present. Note that this sample preparation protocol did not involve solid-phase or liquid extraction and therefore minimized solvent consumption. In addition, the sample preparation steps were simultaneously facile and rapid, enabling a high throughput of samples for the laboratory. 

**Figure 5:**
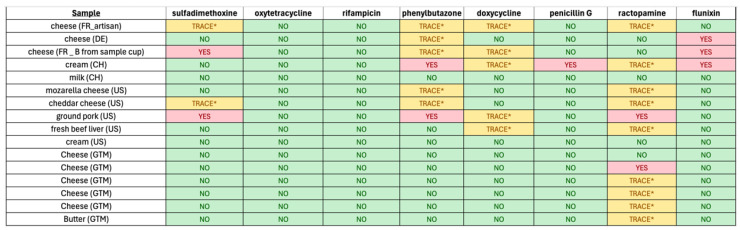
Summary of results of detection of veterinary drug residues present in foods. Note: * Trace indicates a very small peak with properties of the analyte was observed.

Figure 5 reports the results of the analysis of authentic food samples. Neither oxytetracycline nor rifampicin was detected in any of the samples tested, and penicillin G was only indicated as present in cream collected from Switzerland. 

The antibiotics sulfadimethoxine, doxycycline, and nonsteroidal anti-inflammatory phenylbutazone were indicated in trace amounts for selected samples. The nonsteroidal anti-inflammatory drug flunixin was indicated as present within some European dairy products. The animal feed additive, β adrenoreceptor agonist, and the growth enhancer ractopamine appeared to be the most prevalent drugs observed within products tested. Trace levels were indicated, even for selected European samples. This result is unexpected, because the use of ractopamine is banned in the EU and China. Of course, these results are based on insufficient samples to adequately describe the state of the current food supply vis-à-vis veterinary drug residues. However, these results demonstrate that rapid screening of food samples with a minimally laborious and green sample preparation protocol is possible. 

## 4. Discussion

The flow-through dialysis probe sampling method utilized here enables a significant reduction in solvent use while streamlining the sample preparation and reducing the overall analysis time. For instance, labs employing stir-bar extractive sampling generate 10–20 mL of solvent waste per sample [31,46]. Authors employing solid-phase extraction have reported generating ~8–50 mL of waste solvent per sample in their experiments [36,37,38]. The microdialysis sampling approach reduces solvent consumption to only 5–50 microliters per sample, representing a 1000-fold reduction. The approach reflects an advance in green chemistry, owing to the reduced solvent consumption and waste, fewer vials and disposable laboratory consumables, and less time required to process samples. Alternatively, traditional sample preparation strategies may allow for the preconcentration of an analyte within a solid matrix prior to analysis, which is fundamentally not possible at present using the microdialysis approach. 

One exciting future direction for this research would involve integrating the flow-through dialysis sampling approach with the use of a standard autosampler. If the dialysis probe diameter could be made small enough, it is conceivable that a needle punch/probe sampling device could be used to automatically sample from vials. Achieving this would represent an exciting step forward, since it would further enable automated high-throughput analysis. It should be noted that although we constructed our own microdialysis probes, the devices are also commercially available. A combination of autosampling capability and automated sample cleanup prior to chromatography would represent a major breakthrough in the chemical analysis of biological samples.

In addition, future work can be carried out to perform a direct comparison between the microdialysis sampling approach and existing conventional sample preparation methods. A comprehensive comparison of the limit of detection, sensitivity, specificity, total analysis time per sample, dynamic range, and linearity between microdialysis and validated sampling methods should occur. 

## 5. Conclusions

A microvolume dialysis probe has enabled high-throughput chemical analysis while simplifying the sample preparation for veterinary drug detection in complex samples, such as animal product homogenates. When tested with samples that were intentionally spiked with drug residues, the probe coupled with LC-MS correctly identified sample classes with a 96% success rate. An analysis of a limited number of food products from three continents returned positive test results, suggesting that additional efforts should be invested in the analysis of animal-derived food products for veterinary drug residues. The probe requires a perfusion flow of only microliters per minute of solvent, making the approach significantly greener when compared to standard sample processing protocols that may require hundreds of milliliters of solvent and generate laboratory waste (vials, solid-phase extraction materials, etc.). 

## Figures and Tables

**Table 1:** Chromatographic variables observed for veterinary drugs.

Compound	T_ret_ (min)	N (Theoretical Plates) *
sulfadimethoxine	10.82	22,950
oxytetracycline	8.96	6980
rifampicin	10.74	10,100
phenylbutazone	12.96	33,000
doxycycline	9.80	8400
penicillin G	9.60	18,200
ractopamine	9.14	7200
flunixin	11.60	11,750

* All values typical of analysis, N = 16 (t_ret_/W)^2^.

**Table 2:** List of prominent MS1 and MS2 peaks observed for veterinary drugs.

Compound	MS (*m*/*z*)	MS/MS (*m*/*z*)
sulfadimethoxine	311.078	92.0495, 108.0442, 156.0759, 218.0218, 245.1019
oxytetracycline	461.152	154.049, 226.0699, 337.0689, 381.0587, 426.1159
rifampicin	823.405	n.a. *
phenylbutazone	309.157	94.065, 120.044, 146.0592, 160.111, 188.106, 211.085, 253.0956
doxycycline	445.152	126.054, 154.049, 267.0636, 321.0743, 339.0849, 392.110, 410.1214, 428.1319
penicillin G	335.104	128.0523, 160.0418, 176.0697, 289.0987
ractopamine	302.1727	91.054, 107.049, 121.0644, 136.075, 164.1063, 284.1632
flunixin	297.0831	279.0728

* Extensive fragment peaks from rifampicin were not observed in our MS2 experiments.

## Data Availability

The raw data supporting the conclusions of this article will be made available by the authors on request.

## Supplementary Materials

The following supporting information can be downloaded at: https://www.mdpi.com/article/10.3390/s24123971/s1, MS2 Spectra of select veterinary drugs.
