# Supplementary material for: Rapid and Online Microvolume Flow-Through Dialysis Probe for Sample Preparation in Veterinary Drug Residue Analysis
Source: Sensors (Basel). 2024 Jun 19;24(12):3971. doi: 10.3390/s24123971 (PMC11207326; doi:10.3390/s24123971)

D:\antiobtics\Ractopamin_660ug_ml_20240408113340.raw 5/9/2024 12:06:16 PM

Ractopamin_660ug_ml_20240408113340 #3236-3734 RT: 8.16-9.08 AV: 66 NL: 5.65E8
T: FTMS + p NSI d Full ms2 302.1736@hcd30.00 [50.0000-640.0000]

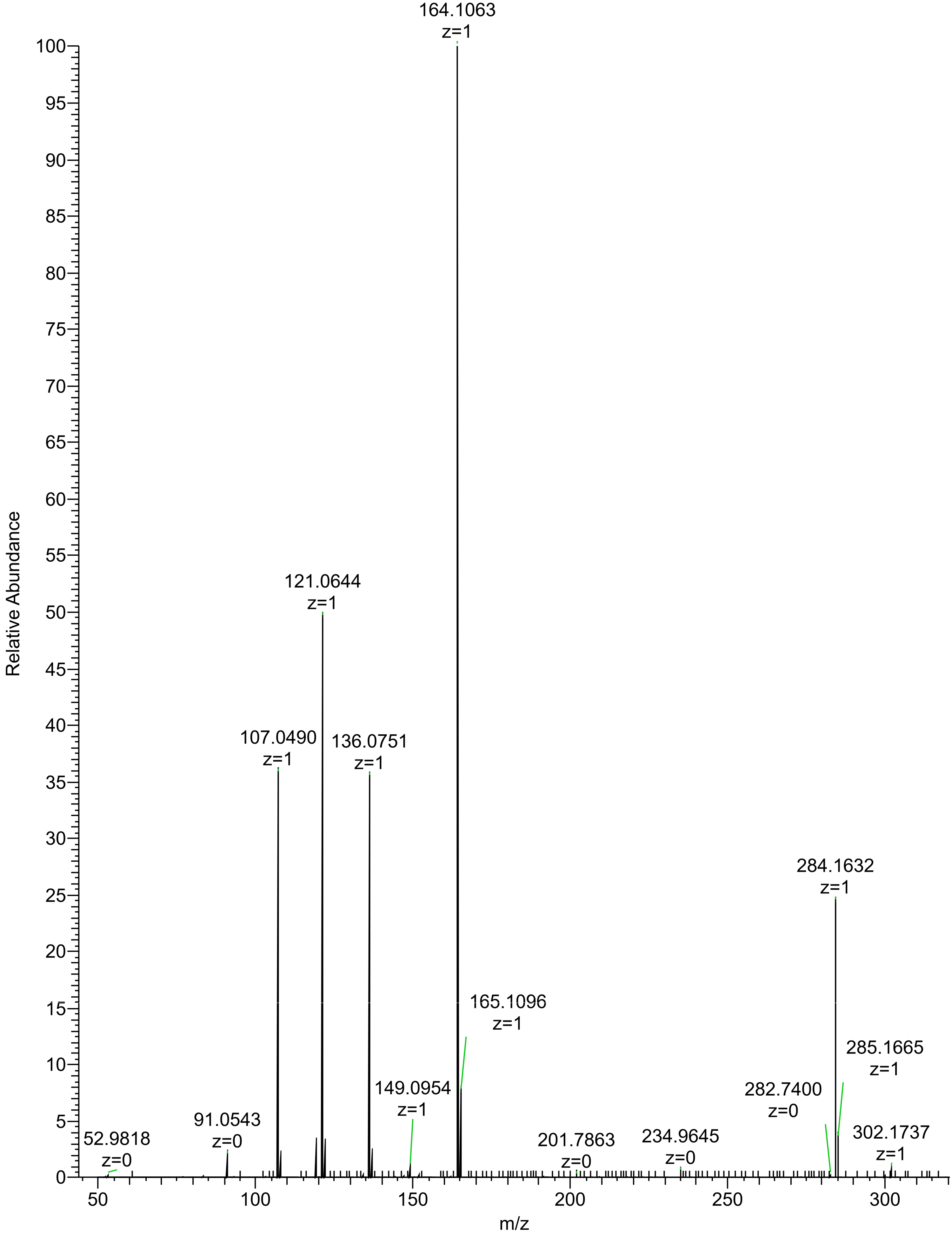

flunixin_1130ug_ml #4769-5334 RT: 11.42-12.54 AV: 5 NL: 5.00E6
T: FTMS + p NSI d Full ms2 297.0826@hcd30.00 [50.0000-325.0000]

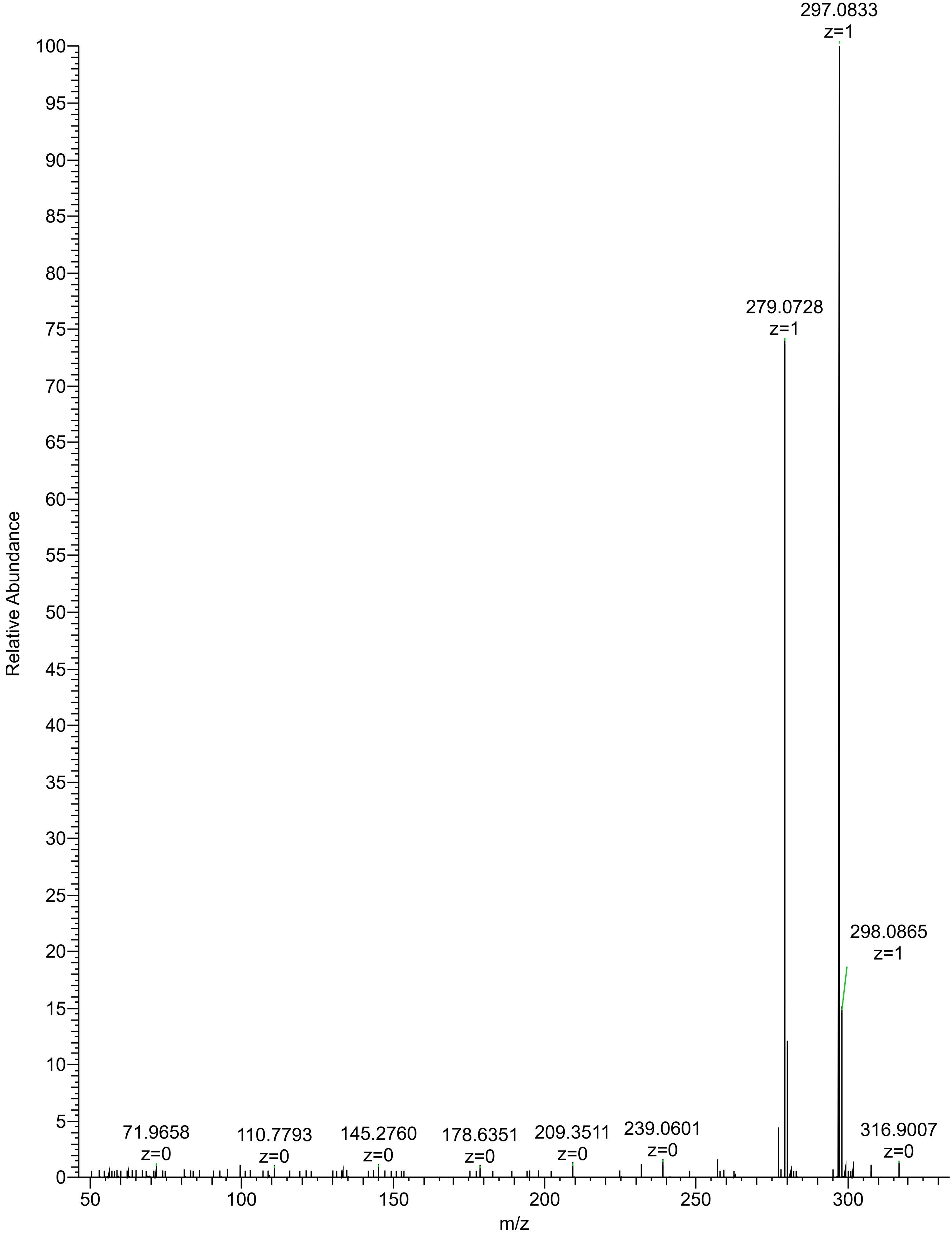

doxycycline_1mg_ml #3625-4650 RT: 9.08-11.02 AV: 8 NL: 8.49E7
T: FTMS + p NSI d Full ms2 445.1585@hcd30.00 [50.0000-475.0000]

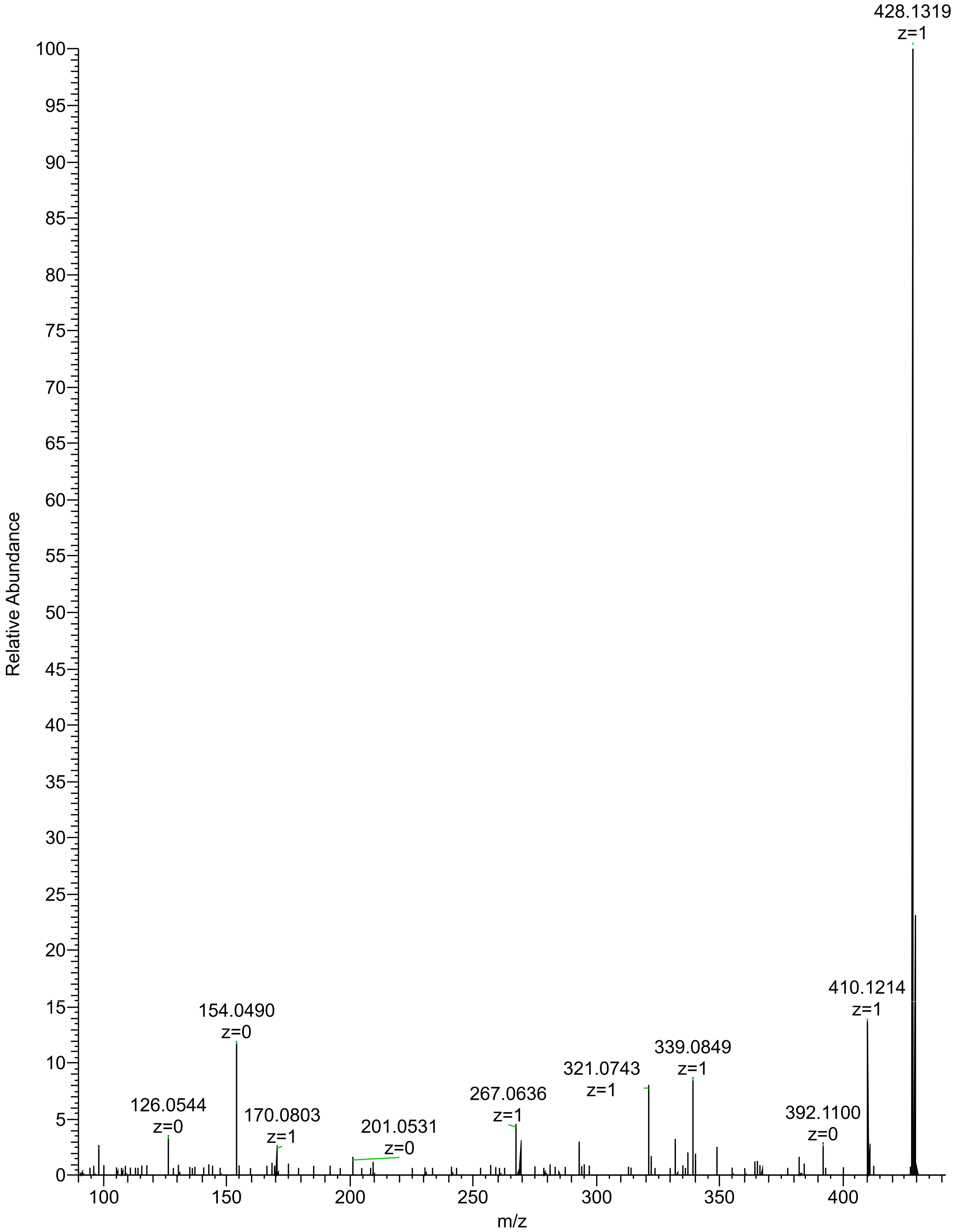

D:\antiobtics\phenylbutazone_0_2mg_ml.raw 5/9/2024 11:56:02 AM

phenylbutazone_0_2mg_ml #5320-5894 RT: 12.71-13.82 AV: 6 NL: 7.31E6
T: FTMS + p NSI d Full ms2 309.1579@hcd30.00 [50.0000-335.0000]

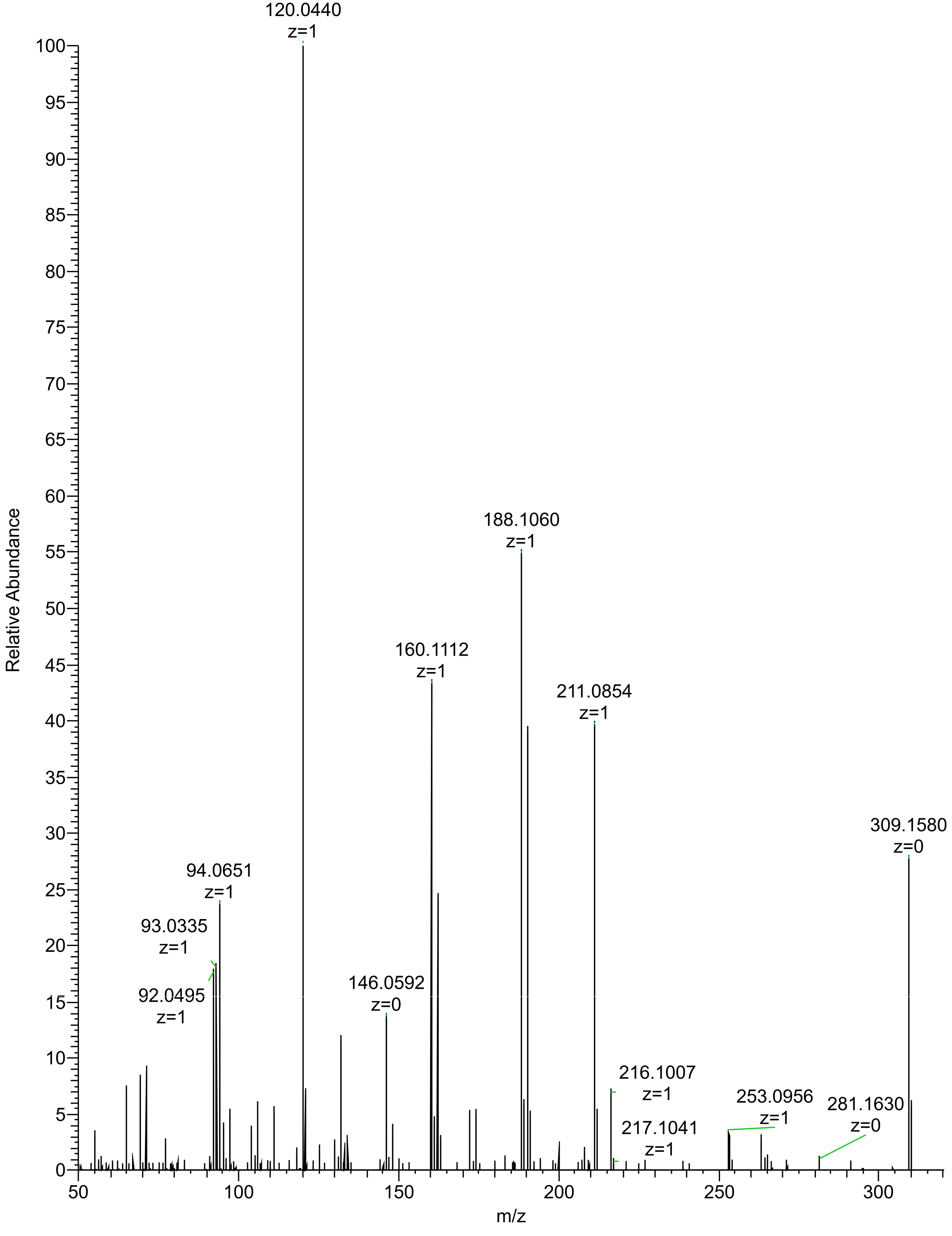

D:\antiobtics\sulfadimethoxine_0_2mg_ml.raw 5/9/2024 11:53:59 AM

sulfadimethoxine_0_2mg_ml #4127-4175 RT: 10.75-10.84 AV: 14 NL: 5.41E8
T: FTMS + p NSI d Full ms2 311.0793@hcd30.00 [50.0000-655.0000]

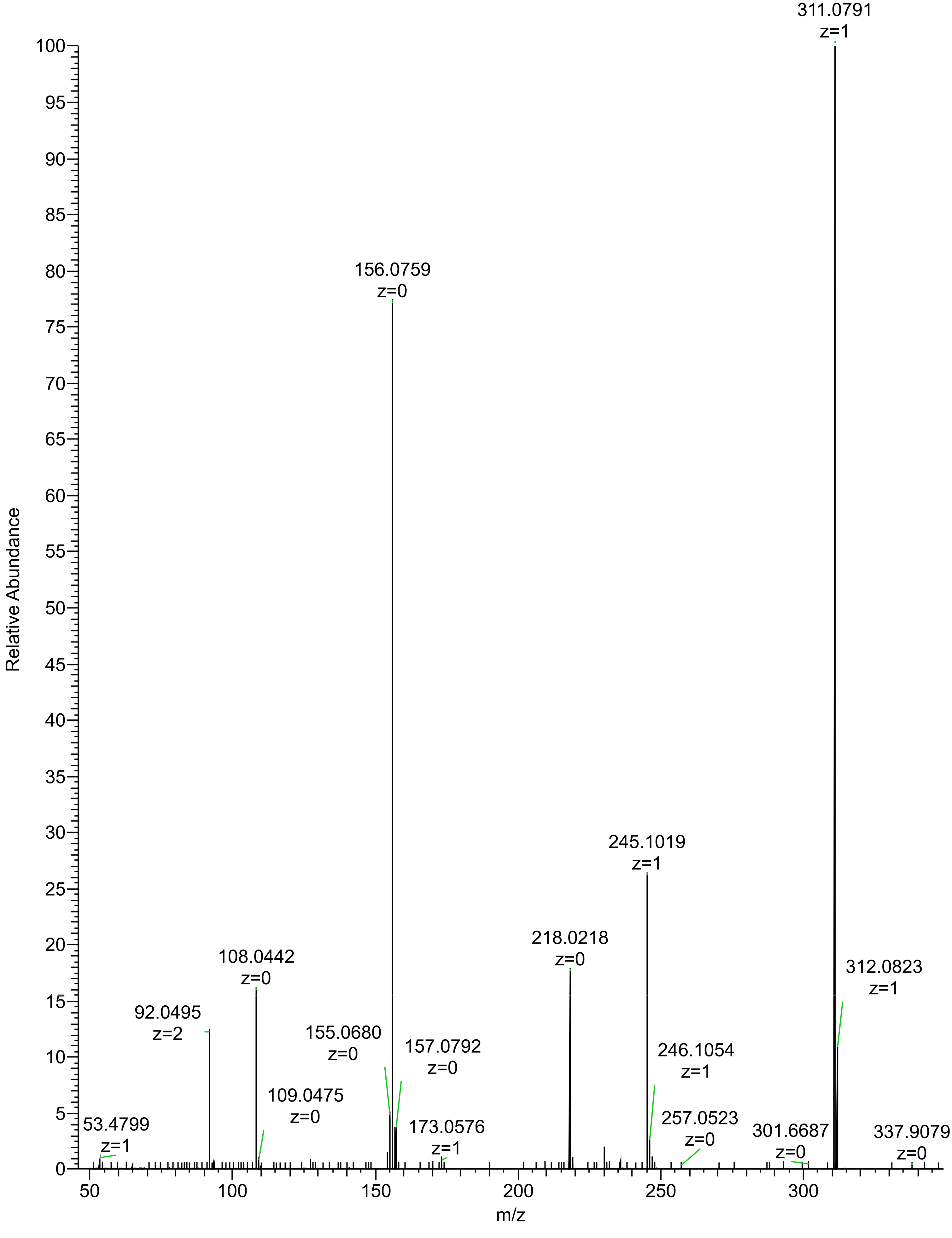

D:\antiobtics\penicilling_5mg_ml.raw 5/9/2024 11:52:09 AM

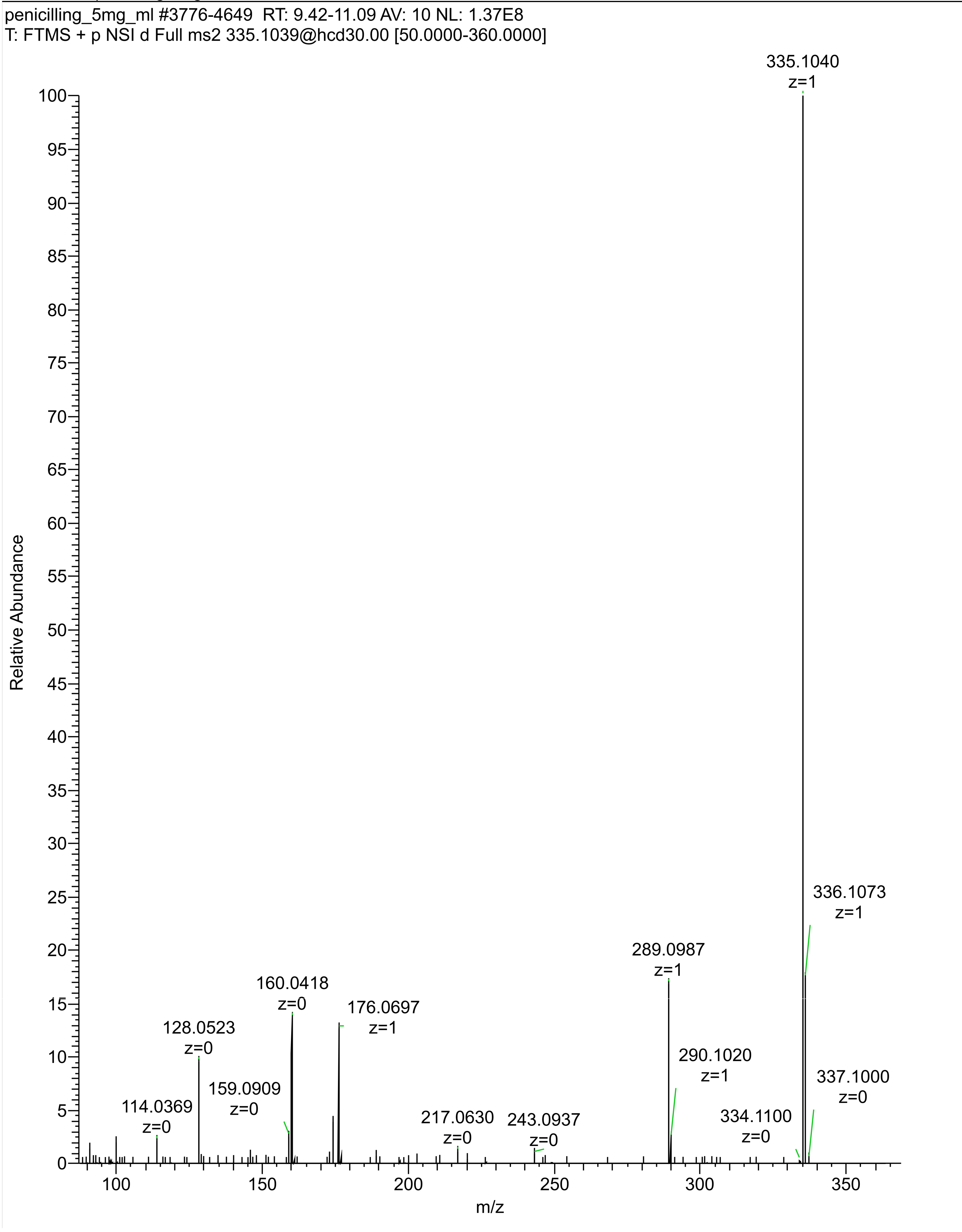

oxytetracycline_1mg_ml #3534-3700 RT: 8.74-9.05 AV: 11 NL: 6.17E8
T: FTMS + p NSI d Full ms2 461.2682@hcd30.00 [64.3333-965.0000]

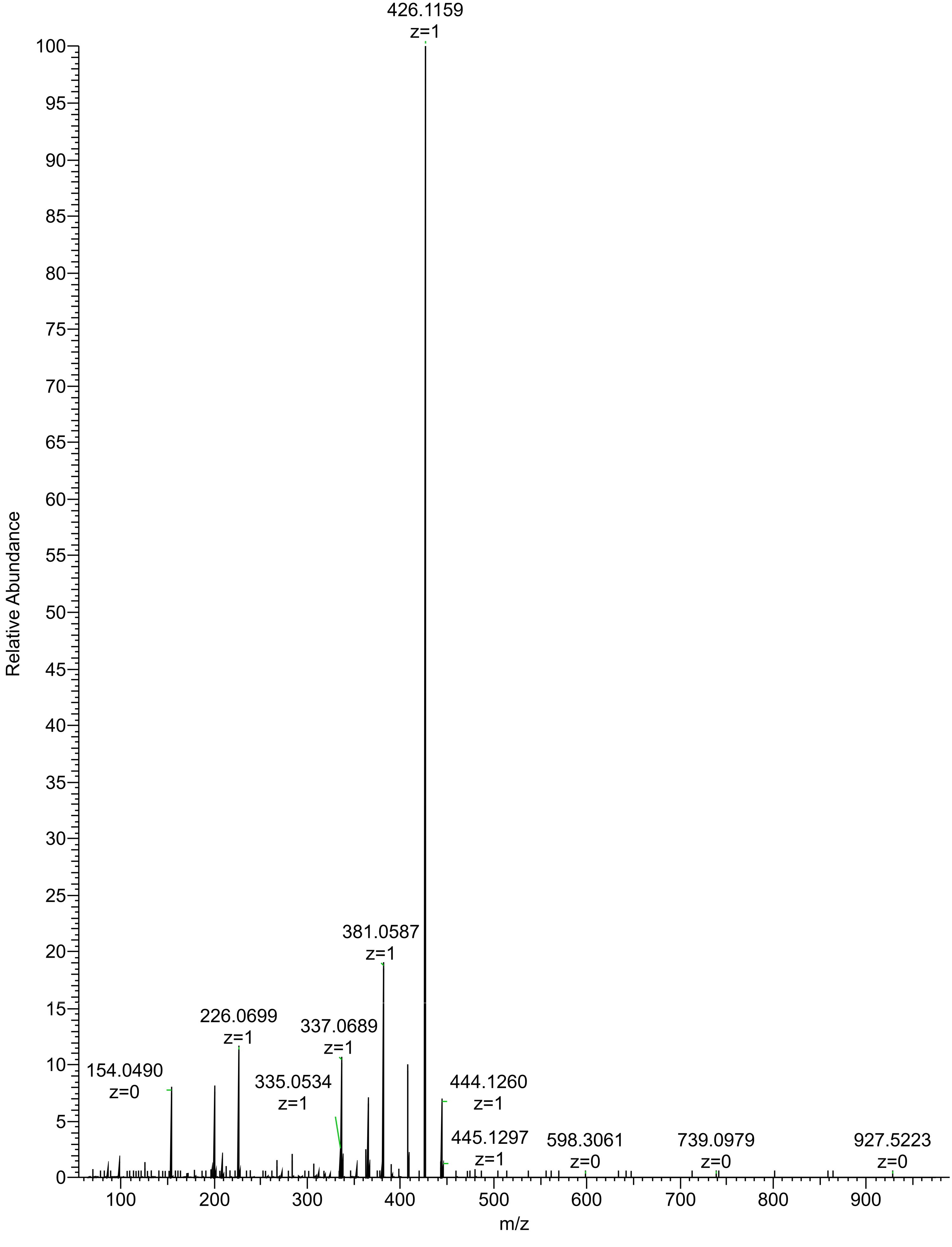

Supplement: Supplementary file 1 [file sensors-24-03971-s001.zip › sensors-3047308-supplementary.pdf]
